# Cacao liquor procyanidins prevent postprandial hyperglycaemia by increasing glucagon-like peptide-1 activity and AMP-activated protein kinase in mice

**DOI:** 10.1017/jns.2018.28

**Published:** 2019-01-16

**Authors:** Yoko Yamashita, Masaaki Okabe, Midori Natsume, Hitoshi Ashida

**Affiliations:** 1 Department of Agrobioscience, Graduate School of Agricultural Science, Kobe University, Nada-ku, Kobe, Hyogo 657-8501, Japan; 2 Food Science & Technology Research laboratories, Meiji Co., Ltd., Hachiouji, Tokyo 192-0919, Japan

**Keywords:** AMP-activated protein kinase, Glucagon-like peptide-1, GLUT4, Hyperglycaemia, Procyanidin, AMPK, AMP-activated protein kinase, CLPr, cacao liquor procyanidin-rich extract, DP, degrees of polymerisation, DPP-4, dipeptidyl peptidase-4 enzyme, GLP-1, glucagon-like peptide-1, IR, insulin receptor, IRS, insulin receptor substrate, OGTT, oral glucose tolerance test

## Abstract

Procyanidins have been reported to possess potential for the prevention of hyperglycaemia. However, there are very few data for procyanidins about the difference the degree of polymerisation (DP) has on anti-hyperglycaemic effects. Moreover, the underlying molecular mechanisms by which procyanidins suppress hyperglycaemia are not yet fully understood. In the present study, we prepared procyanidin fractions with different DP, namely low-DP (DP≤3) and high-DP (DP≥4) fractions, from a cacao liquor procyanidin-rich extract (CLPr). These fractions were administered orally to Institute of Cancer Research (ICR) mice and their anti-hyperglycaemic effects were examined. We found that CLPr and its fractions prevent postprandial hyperglycaemia accompanied by an increase in the plasma glucagon-like peptide-1 (GLP-1) level with or without glucose load. In the absence of glucose load, both fractions increased the plasma insulin level and activated its downstream signalling pathway in skeletal muscle, resulting in promotion of the translocation of GLUT4. Phosphorylation of AMP-activated protein kinase (AMPK) was also involved in the promotion of GLUT4 translocation. High- and low-DP fractions showed a similar activation of insulin and AMPK pathways. In conclusion, cacao liquor procyanidins prevent hyperglycaemia by promoting GLUT4 translocation in skeletal muscle, and both the GLP-1-activated insulin pathway and the AMPK pathway are involved in the underlying molecular mechanism.

Long-term persistence of hyperglycaemia increases the risk of specific complications and CVD^(^^1^^)^. Glucoregulatory hormones including insulin, glucagon, glucagon-like peptide-1 (GLP-1), glucose-dependent insulinotropic peptide (GIP), adrenalin and cortisol maintain circulating glucose concentration within a relatively narrow range^(^^2^^)^. Of these, GLP-1 and GIP are known as incretin hormones^(^^3^^)^. Although insulin is a key regulatory hormone of glucose disappearance, current therapeutic agents to treat type 2 diabetes mellitus are focused on compounds possessing incretin effects.

Incretin hormones augment the magnitude of meal-stimulated insulin secretion from pancreatic β-cells in a glucose-dependent manner^(^^2^^)^. The dominant incretin hormones, GLP-1 and GIP, are associated with glucose homeostasis. Intravenous administration of GLP-1 ameliorates hyperglycaemia in patients with type 2 diabetes mellitus^(^^3^^)^. However, GLP-1 has an extremely short half-life because of its rapid catabolism by the enzyme dipeptidyl peptidase-4 (DPP-4)^(^^4^^)^. GLP-1 receptor agonists^(^^5^^)^, GLP-1 analogues^(^^3^^)^ and DPP-4 inhibitors^(^^6^^)^ are, therefore, being developed as therapeutic agents to treat type 2 diabetes mellitus. Recently, certain non-nutrient food components and extracts have been reported to possess incretin effects. For example, a grape seed procyanidin extract and chalcone have inhibitory effects on DPP-4^(^^7^^,^^8^^)^, and intake of resveratrol for 5 weeks enhanced GLP-1 secretion when combined with an oral glucose load in high-fat diet-fed mice^(^^9^^)^. These results strongly suggest that polyphenols have the potential to modulate GLP-1 levels. We also reported that cinnamtannnin A2, a tetramer procyanidin, increased GLP-1 secretion 60 min after oral administration^(^^10^^)^. It is, however, unknown whether other procyanidins increase endogenous GLP-1 secretion and their underling mechanisms.

Procyanidins, flavan-3-ol oligomers and polymers, are widely distributed throughout the plant kingdom. Among edible plants, they are rich in cacao, black soyabean, cinnamon and apple. Of these, the anti-hyperglycaemic effects of cacao procyanidins are well documented. For example, daily intake of cocoa or dark chocolate has the potential to prevent diabetes mellitus, hyperglycaemia and CVD^(^^11^^–^^13^^)^. Our previous reports^(^^14^^,^^15^^)^ demonstrate that a cacao liquor procyanidin-rich extract (CLPr) prevented hyperglycaemia and obesity through activation of AMP-activated protein kinase (AMPK)α, promoting GLUT4 translocation and enhancing uncoupling protein (UCP)3 and PGC1α expressions in skeletal muscle of mice^(^^15^^)^. We subsequently prepared procyanidin-rich fractions from CLPr based on degree of polymerisation (DP) and found that both high-DP (DP≥4) and low-DP (DP≤3) fractions increased glucose uptake activity and GLUT4 translocation through the AMPK-dependent pathway in L6 myotubes^(^^16^^)^. In the present study, we confirmed an anti-hyperglycaemic effect of high- and low-DP fractions in Institute of Cancer Research (ICR) mice using an oral glucose tolerance test (OGTT), and then investigated whether these procyanidin-rich fractions increased plasma GLP-1. Our findings suggest a novel molecular mechanism that procyanidin-caused anti-hyperglycaemic effects were due to both GLP-1 secretion and AMPK activation.

## Materials and methods

### Materials

K597, a DPP-4 inhibitor, and the Wako Labassay™ Glucose kit were from Wako Pure Chemical. Primary antibodies against GLUT1 and GLUT4, horseradish peroxidase-conjugated anti-goat, anti-rabbit and anti-mouse IgG antibodies and protein A/G plus-agarose were obtained from Santa Cruz Biotechnology Inc. Anti-AMPKα, anti-PI3K, anti-Akt and anti-insulin receptor (IR)β were from Cell Signaling Technology Inc. Anti-phosphotyrosine and anti-insulin receptor substrate (IRS)-1 were from Becton, Dickinson and Company. All other reagents used were of the highest grade available from commercial sources.

### Cacao liquor polyphenols

CLPr was prepared from cacao liquor as previously described^(^^15^^)^. The total polyphenol content in CLPr was 69·8 %. Each polyphenol in CLPr was quantified by HPLC and liquid chromatography–MS^(^^17^^,^^18^^)^. The amount of each procyanidin is represented as epicatechin equivalents. CLPr consisted of 4·28 % (+)-catechin, 6·12 % (−)-epicatechin, 3·60 % procyanidin B2, 0·75 % procyanidin B5, 2·28 % procyanidin C1 and 1·01 % cinnamtannin A2. Low-DP (DP≤3) and high-DP (DP≥4) procyanidin fractions were separated from CLPr as previously described^(^^19^^)^. Their total polyphenol contents were 75·4 % and 66·1 %, respectively. The procyanidin composition of the low-DP fraction was 10·03 % (+)-catechin, 14·56 % (−)-epicatechin, 8·43 % procyanidin B2, 1·79 % procyanidin B5, 5·25 % procyanidin C1 and 0·23 % cinnamtannin A2, while that of the high-DP fraction was 0·25 % (+)-catechin, 0·39 % (−)-epicatechin, 0·24 % procyanidin B2, 0·31 % procyanidin C1 and 2·91 % cinnamtannin A2. The remaining were other oligomeric and polymeric procyanidins in all three fractions.

### Animal treatments

All animal experiments were approved by the Institutional Animal Care and Use Committee (permission no. 24-04-02) and carried out according to the guidelines for animal experiments at Kobe University. Male Jcl:ICR mice (4 weeks old) were obtained from Japan SLC Inc. and were intercrossed to obtain a closed colony. The mice were maintained in a temperature-controlled room (23±2°C) with a 12 h–12-h light–dark cycle (lights on at 09.00 hours). The mice were acclimatised for 7 d with free access to a commercial standard mouse diet consisting of 76 % carbohydrate, 15 % protein and 9 % fat (3·850 kcal (16·108 kJ)/g diet; Research Diets) and distilled water (see Table 1). These mice were used for the following experiments.

**Table 1.** Composition of diet

	Composition (g/kg diet)
Casein	140
l-Cystine	1·8
Maize starch	495·692
Maltodextrin	125
Sucrose	100
Cellulose	50
Soyabean oil	40
*tert*-Butylhydroquinone	0·008
AIN-93 mineral mixture	35
AIN-93 vitamin mixture	10
Choline bitartrate	2·5

AIN, American Institute of Nutrition.

#### Experiment 1

For the OGTT, thirty mice were divided into six groups of five each. CLPr at 10 mg/kg body weight, high- or low-DP fractions at 1·0 and 10 mg/kg body weight, or water alone (5 ml/kg body weight) as a vehicle control were orally administered to the mice after 18 h fasting. At 60 min after the administration, mice received an oral glucose dose of 1·0 g/kg body weight. Blood was collected from the tail vein in heparinised tubes at −60 (before sample administration), 0 (before glucose load), 15, 30, 60 and 120 min after the glucose load and centrifuged at 9600 ***g*** for 10 min at 4°C for preparation of the plasma^(^^14^^)^. Plasma was collected and subjected to the measurement of the glucose level using the commercial kit.

To measure GLUT4 translocation and its related signalling pathways, another twenty mice were randomly assigned to four groups of five each. They were given an oral dose of either CLPr, or the high- or low-DP fractions in water at 10 mg/kg body weight after 18 h fasting. Mice in the control group received water alone (5 ml/kg body weight). The mice were killed 60 min after the oral administration of CLPr and its fractions under anaesthesia using sevoflurane as an inhalational anaesthetic and sodium pentobarbital as an analgesic. The soleus muscle was collected from hind legs and its plasma membrane fraction and tissue lysate were prepared as previously described^(^^20^^)^. The small intestine was also collected and α-glucosidase activity measured as described in a previous report^(^^14^^)^.

#### Experiment 2

For the measurement of GLP-1 secretion, sixty mice were randomly assigned to twenty groups of five mice each (four groups for each five time points). There were orally administered CLPr and its fractions (low-DP and high-DP) in water at 10 mg/kg body weight, or water alone (5 ml/kg body weight) as a vehicle control, and then killed under anaesthesia using sodium pentobarbital, and euthanised by exsanguination from cardiac puncture at 0, 15, 30, 45 and 60 min. Another 100 mice were randomly assigned to twenty groups of five mice each (four groups for each five time points). They were orally administered CLPr and its fractions (low-DP and high-DP) in water at 10 mg/kg body weight, or water alone (5 ml/kg body weight) as a vehicle control. At 60 min after the administration, mice received an oral glucose dose of 1·0 g/kg body weight. Mice were killed under anaesthesia using sevoflurane as an inhalational anesthetic and sodium pentobarbital as an analgesic, and euthanised by exsanguination from cardiac puncture at −60, 0, 5, 15 and 30 min after the glucose load. The blood sample was collected to a plastic tube containing the DPP-4 inhibitor K597. Plasma was prepared by centrifuging the blood at 9600 ***g*** for 10 min at 4°C^(^^14^^)^. Measurements of plasma GLP-1 (7-36 amid) and insulin levels were performed using the corresponding ELISA kit at the time points indicated in Figs 6 and 7, respectively.

### Immunoprecipitation and immunoblotting

Immunoblotting was performed according to our previous method^(^^21^^)^. Briefly, proteins in the muscle plasma membrane and tissue lysate were separated using sodium dodecyl sulfate (SDS) polyacrylamide gel and transferred to a polyvinylidene difluoride membrane. After blocking with Blocking One solution (Nacalai Tesque), the membrane was incubated with the specified primary antibody (1:5000) overnight at 4°C, followed by the corresponding horseradish peroxidase-conjugated secondary antibody (1:20 000) for 1 h at room temperature. The proteins were visualised using the ImmunoStar^®^ LD luminescence system (Wako Pure Chemical Industries Ltd) and detected using a Light-Capture II imaging system (ATTO Corp.).

Immunoprecipitation was performed as previously described^(^^21^^)^ with slight modifications. In brief, an aliquot of muscle lysate fraction containing 300 µg of protein was incubated with 20 µl protein A/G plus-agarose for 1 h at 4°C. The mixture was centrifuged at 1000 ***g*** for 10 min at 4°C, and the resultant supernatant fraction was transferred to a new tube. The supernatant fraction was incubated overnight at 4°C with antibodies specific to PY20. After adding 30 µl of protein A/G plus-agarose beads and incubating for 90 min at 4°C, the beads containing immune complexes were centrifuged at 1000 ***g*** for 10 min at 4°C and washed five times with PIPA buffer (10 mm-Tris, pH 8·0, 150 mm-sodium chloride, 0·5 % (w/v) sodium deoxycholate, 0·1 % (w/v) SDS, 1·0 % (v/v) Nonidet P-40 and 0·5 mm-dithiothreitol) containing protease and phosphatase inhibitors (1 mm-phenylmethylsulfonyl fluoride, 5 µg/ml leupeptin, 5 µg/ml aprotinin, 10 mm-NaF and 1 mm-Na_3_VO_4_). The immune complexes were suspended in 2 × SDS buffer, boiled for 5 min, and centrifuged at 1000 ***g*** for 2 min. The supernatant fraction was subjected to immunoblotting with specific antibodies against IRβ (1:1000) or IRS-1 (1:1000), followed by the corresponding horseradish peroxidase-conjugated secondary antibody (1:20 000).

### Measurement of α-glucosidase activity in the small intestine

α-Glucosidase activity was measured in the small intestine of mice as previously described^(^^14^^)^. The intestinal mucosa was collected and homogenised with three volumes of 1·15 % KCl solution on ice. The homogenate was centrifuged at 1000 ***g*** for 10 min at 4°C, and the resultant supernatant fraction was used for the measurements of maltase and sucrase–isomaltase activities^(^^14^^)^.

### Statistical analysis

**Fig. 1.**
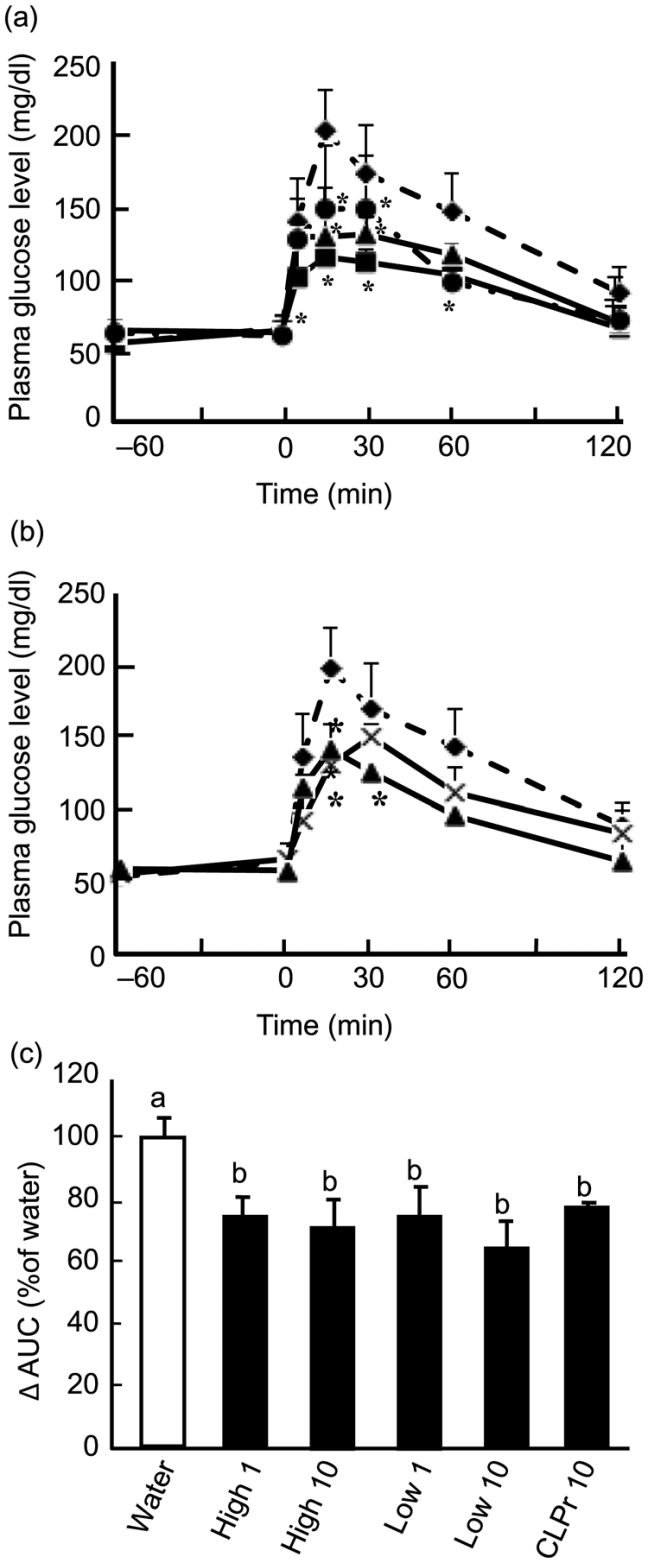
Effects of cacao liquor procyanidin-rich extract (CLPr) and its fractions on plasma glucose levels in the oral glucose tolerance test. Institute of Cancer Research (ICR) mice were orally dosed with the procyanidin fractions (1·0 or 10 mg/kg body weight), CLPr or water (vehicle control; 5 ml/kg body weight). At 60 min after administration, mice received an oral glucose solution (1·0 g/kg body weight), and the plasma glucose levels were measured −60 min (before administration of CLPr and its fractions), 0 min (just before administration of glucose), and 5, 15, 30, 60 and 120 min after glucose administration. Results of the oral glucose tolerance tests are shown after treatment with 10 (A) or 1·0 (B) mg/kg body weight of high-degree of polymerisation (DP) fraction (■), low-DP fraction (▲), CLPr (●) or water (♦). The AUC was calculated from the results in (A) and (B) and is shown in (C). Values are means (*n* 5), with standard errors represented by vertical bars. * Mean value was significantly different from that of the corresponding control group (*P* < 0·05; Dunnett's test in (A) and (B)). ^a,b^ Mean values with unlike letters were significantly different (*P* < 0·05; Tukey–Kramer multiple comparison test in (C)). To convert glucose in mg/dl to mmol/l, multiply by 0·0555.

**Fig. 2.**
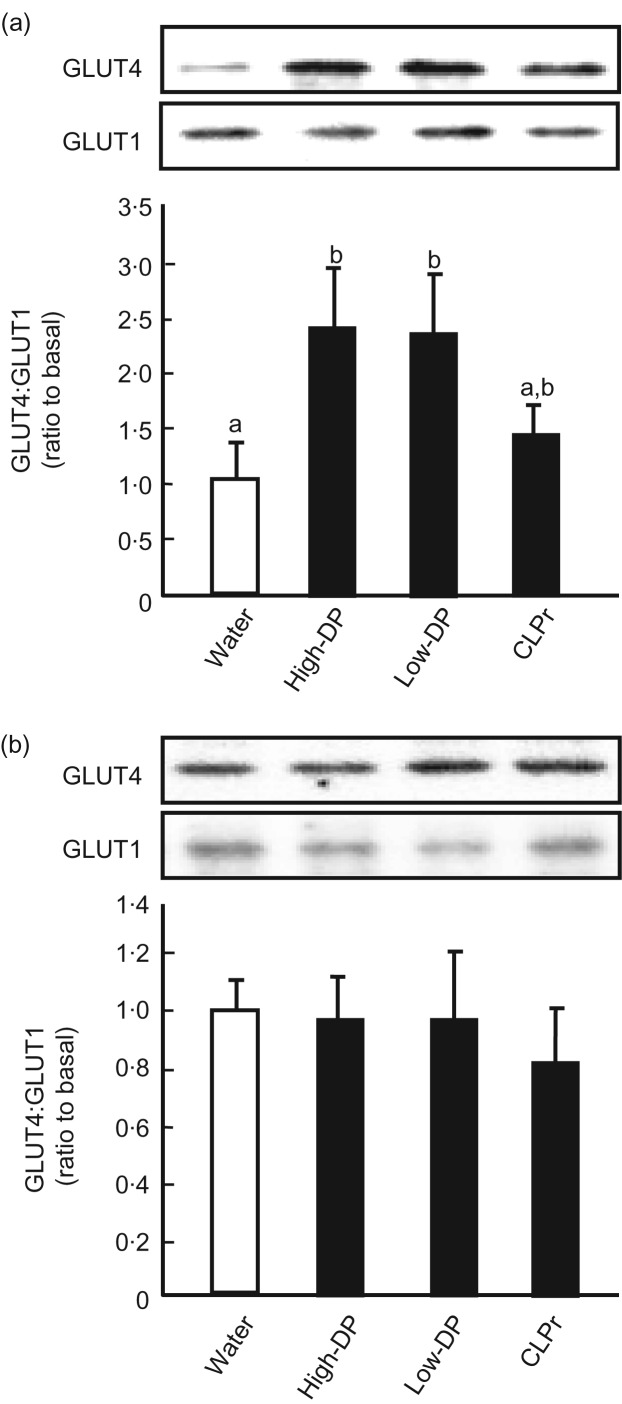
Effects of cacao liquor procyanidin-rich extract (CLPr) and its fractions on GLUT4 translocation in mouse skeletal muscle. Institute of Cancer Research (ICR) mice were orally dosed with 10 mg/kg body weight of procyanidin fractions, CLPr or water as a vehicle control (5 ml/kg body weight), and skeletal muscle was collected 60 min after administration. The amounts of GLUT4 and GLUT1 proteins in the plasma membrane (A) and tissue lysate (B) of the skeletal muscle were determined by immunoblotting. Each panel shows a typical result from four animals. The density of each band was analysed and normalised to that of GLUT1. Values are means (*n* 5), with standard errors represented by vertical bars. ^a,b^ Mean values with unlike letters were significantly different (*P* < 0·05; Tukey–Kramer multiple comparison test). DP, degree of polymerisation.

Data are represented as means with their standard errors. The statistical significance of experimental observations was determined using Dunnett's multiple comparison test (Figs 1 and 2) or the Tukey–Kramer multiple comparison test (all other figures). The level of significance was set at *P* < 0·05.

## Results

### Effects of cacao liquor procyanidin-rich extract on plasma glucose response in an oral glucose tolerance test

We performed an OGTT to evaluate the effect of CLPr and its fractions on postprandial hyperglycaemia. Plasma glucose levels in the control group (glucose alone) increased in response to oral glucose load, reaching a peak at 15 min, and then decreased with time. Following the glucose load, CLPr at 10 mg/kg body weight significantly suppressed the transient increase in plasma glucose levels at 15 min (Fig. 1(A)). The high-DP fraction showed significant suppression of the plasma glucose levels from 5 to 60 min, while the low-DP fraction reduced plasma glucose at 15 and 30 min. When these fractions were administered at 1·0 mg/kg body weight, the high-DP fraction significantly suppressed the plasma glucose level at 15 min, and the low-DP fraction significantly suppressed plasma glucose at 15 and 30 min (Fig. 1(B)). The AUC of the plasma glucose levels is shown in Fig. 1(C). High- and low-DP procyanidin fractions suppressed the acute elevation in plasma glucose levels after glucose load to the same extent.

It is known that an inhibition of α-glucosidase activity contributes to the prevention of postprandial hyperglycaemia. However, we did not observe any significant inhibitory effect of CLPr or its fractions on maltase or sucrose–isomaltase activities in the intestine of mice under our experimental conditions (data not shown).

### High- and low-degree of polymerisation procyanidin fractions promote GLUT4 translocation to the plasma membrane in skeletal muscle

We examined whether oral administration of procyanidin fractions promoted GLUT4 translocation in the plasma membrane. As shown in Fig. 2(A), both high- and low-DP procyanidin fractions significantly promoted GLUT4 translocation in the plasma membrane of skeletal muscle. The expression level of GLUT4 did not alter after administration of CLPr and its fractions (Fig. 2(B)). Moreover, the level of GLUT1 in both the plasma membrane and cell lysate showed no change. These results indicate that the procyanidin fractions decreased postprandial hyperglycaemia through promotion of GLUT4 translocation in skeletal muscle.

### High- and low-degree of polymerisation procyanidin fractions activate the AMP-activated protein kinase signalling pathway in skeletal muscle

**Fig. 3.**
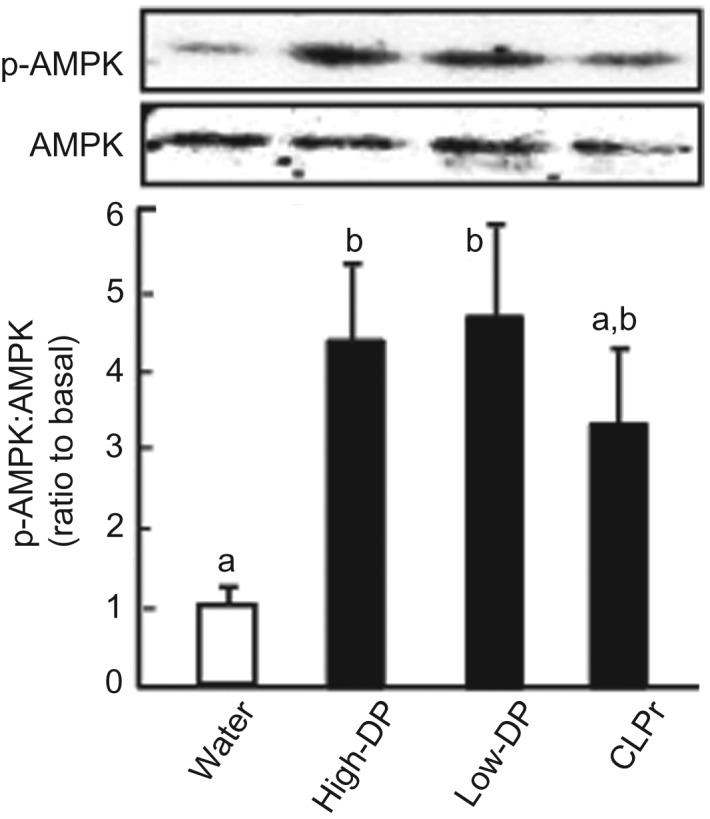
Effects of cacao liquor procyanidin-rich extract (CLPr) and its fractions on AMP-activated protein kinase (AMPK) phosphorylation in skeletal muscle of mice. Institute of Cancer Research (ICR) mice were orally dosed with 10 mg/kg body weight of procyanidin fractions, CLPr or water as a vehicle control (5 ml/kg body weight), and skeletal muscle was collected 60 min after the administration. Tissue lysate of skeletal muscle was subjected to immunoblotting analysis to determine AMPK and its phosphorylated form (p-AMPK). Each panel shows a typical result from four animals. The density of each band was analysed and ratio of phosphorylation level to the expression is shown in the bottom subfigure. Values are means (*n* 5), with standard errors represented by vertical bars. ^a,b^ Mean values with unlike letters were significantly different (*P* < 0·05; Tukey–Kramer multiple comparison test). DP, degree of polymerisation.

It is known that the AMPK-signalling pathway is involved in GLUT4 translocation. Our previous report demonstrated that CLPr increased phosphorylation of AMPK in L6 myotubes^(^^14^^)^ and in muscle of mice fed normal and high-fat diets for 13 weeks^(^^15^^)^. The activation of AMPK was confirmed in skeletal muscle under the present experimental conditions: high- and low-DP procyanidin fractions significantly increased phosphorylation of AMPK (Fig. 3) and CLPr also tended to increase this phosphorylation. In contrast, the expression level of AMPK did not alter in response to any of the treatments. Thus, oral intake of high- and low-DP procyanidin fractions promote GLUT4 translocation by activating the AMPK pathway.

### High- and low-degree of polymerisation procyanidin fractions activate the insulin-signalling pathway in skeletal muscle

**Fig. 4.**
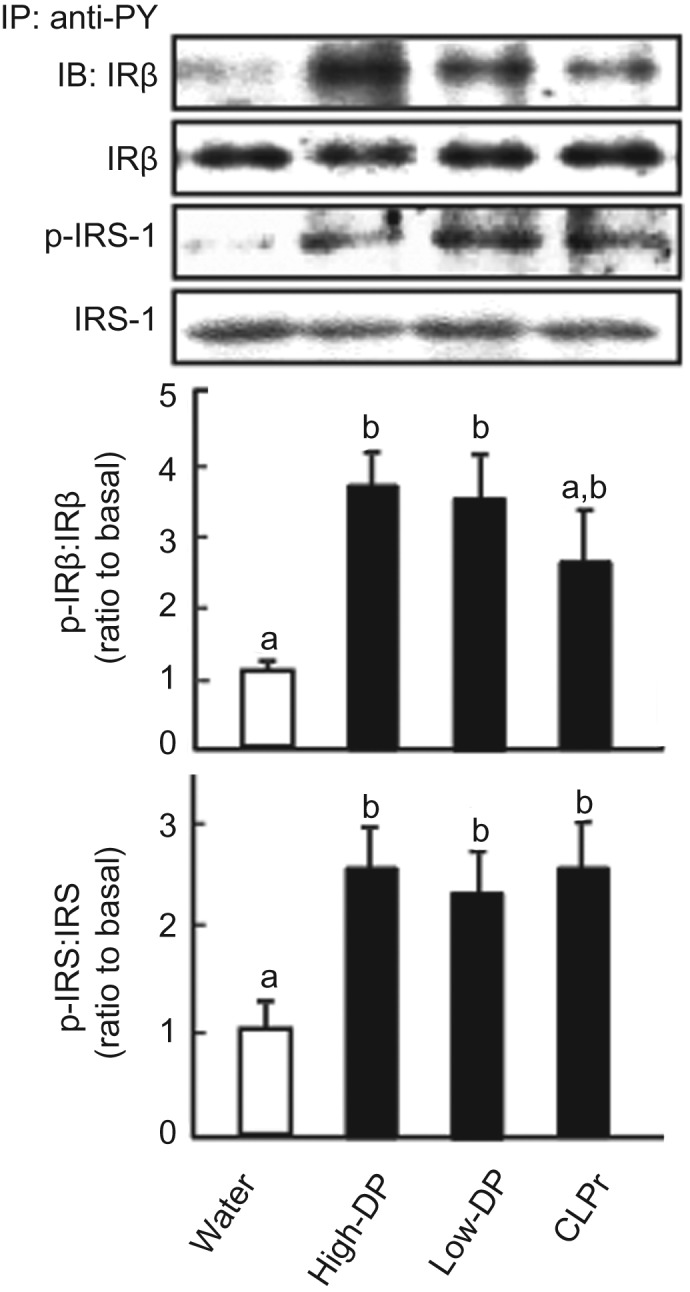
Effect of cacao liquor procyanidin-rich extract (CLPr) and its fractions on phosphorylation of insulin receptor (IR)β and insulin receptor substrate-1 (IRS-1) in mouse skeletal muscle. Institute of Cancer Research (ICR) mice were orally dosed with 10 mg/kg body weight of procyanidin fractions, CLPr or water as a vehicle control (5 ml/kg body weight), and skeletal muscle was collected 60 min after administration. Tissue lysate of skeletal muscle was subjected to immunoblotting analysis to determine IRβ and IRS-1 and their phosphorylated forms (p-IRβ and p-IRS-1). Each panel shows a typical result from four animals. The density of each band was analysed and ratios of phosphorylation level to the expression are shown in the middle and bottom subfigures. Values are means (*n* 5), with standard errors represented by vertical bars. ^a,b^ Mean values with unlike letters were significantly different (*P* < 0·05; Tukey–Kramer multiple comparison test). DP, degree of polymerisation; IP, immunoprecipitation; PY, phosphotyrosine; IB, immunoblot.

It is known that the insulin-signalling pathway is also mainly involved in GLUT4 translocation. Thus, we investigated the effect of CLPr and its fractions on the phosphorylation of IRβ, IRS-1, PI3K and Akt (which are members of the insulin-signalling pathway) in skeletal muscle of mice under the same treatment conditions as those described for Fig. 2. Both procyanidin fractions significantly increased phosphorylation of IRβ and IRS-1 in muscle compared with that in the muscle of control mice (Fig. 4). In the case of CLPr, phosphorylation of IRS-1 was significantly increased and phosphorylation of IRβ showed a tendency to increase.

**Fig. 5.**
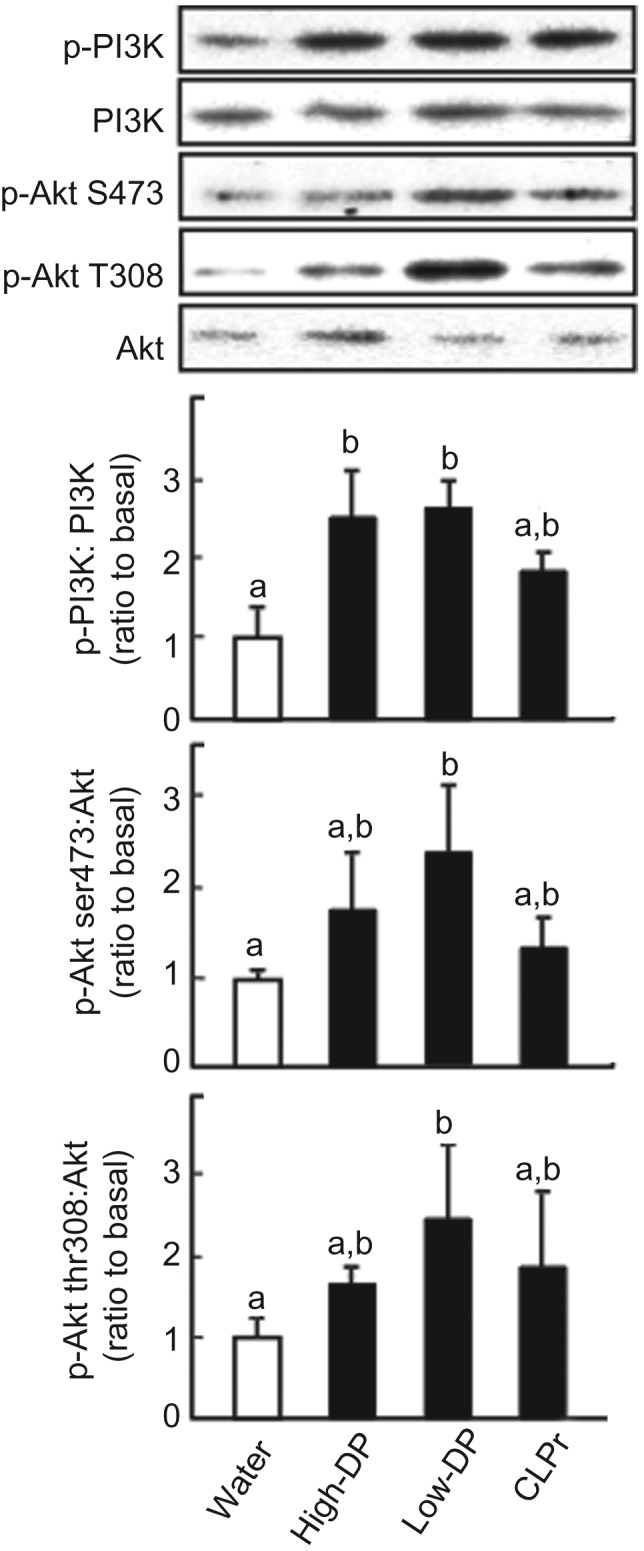
Effect of cacao liquor procyanidin-rich extract (CLPr) and its fractions on phosphorylation of phosphoinositide 3-kinase (PI3K) and protein kinase B (Akt) in mouse skeletal muscle. Institute of Cancer Research (ICR) mice were orally dosed with 10 mg/kg body weight of procyanidin fractions, CLPr or water as a vehicle control (5 ml/kg body weight), and skeletal muscle was collected 60 min after administration. Tissue lysate of skeletal muscle was subjected to immunoblotting analysis to determine PI3K and Akt and their phosphorylation forms (p-PI3K, p-Akt at ser473 and thr308). Each panel shows a typical result from four animals. The density of each band was analysed and ratios of phosphorylation level to the expression are shown the bottom three subfigures. Values are means (*n* 5), with standard errors represented by vertical bars. ^a,b^ Mean values with unlike letters were significantly different (*P* < 0·05; Tukey–Kramer multiple comparison test). DP, degree of polymerisation.

Regarding PI3K as a downstream kinase of IRS-1, both high- and low-DP procyanidin fractions significantly promoted the phosphorylation of PI3K (Fig. 5). The low-DP fraction significantly increased phosphorylation of Akt at Ser473 and Thr 308, while the high-DP fraction and the original CLPr showed a tendency to promote the phosphorylation of Akt. These results indicate that procyanidin fractions promote GLUT4 translocation accompanied by both AMPK and insulin-signalling pathways.

### High- and low-degree of polymerisation procyanidin fractions increase glucagon-like peptide-1 secretion in plasma

**Fig. 6.**
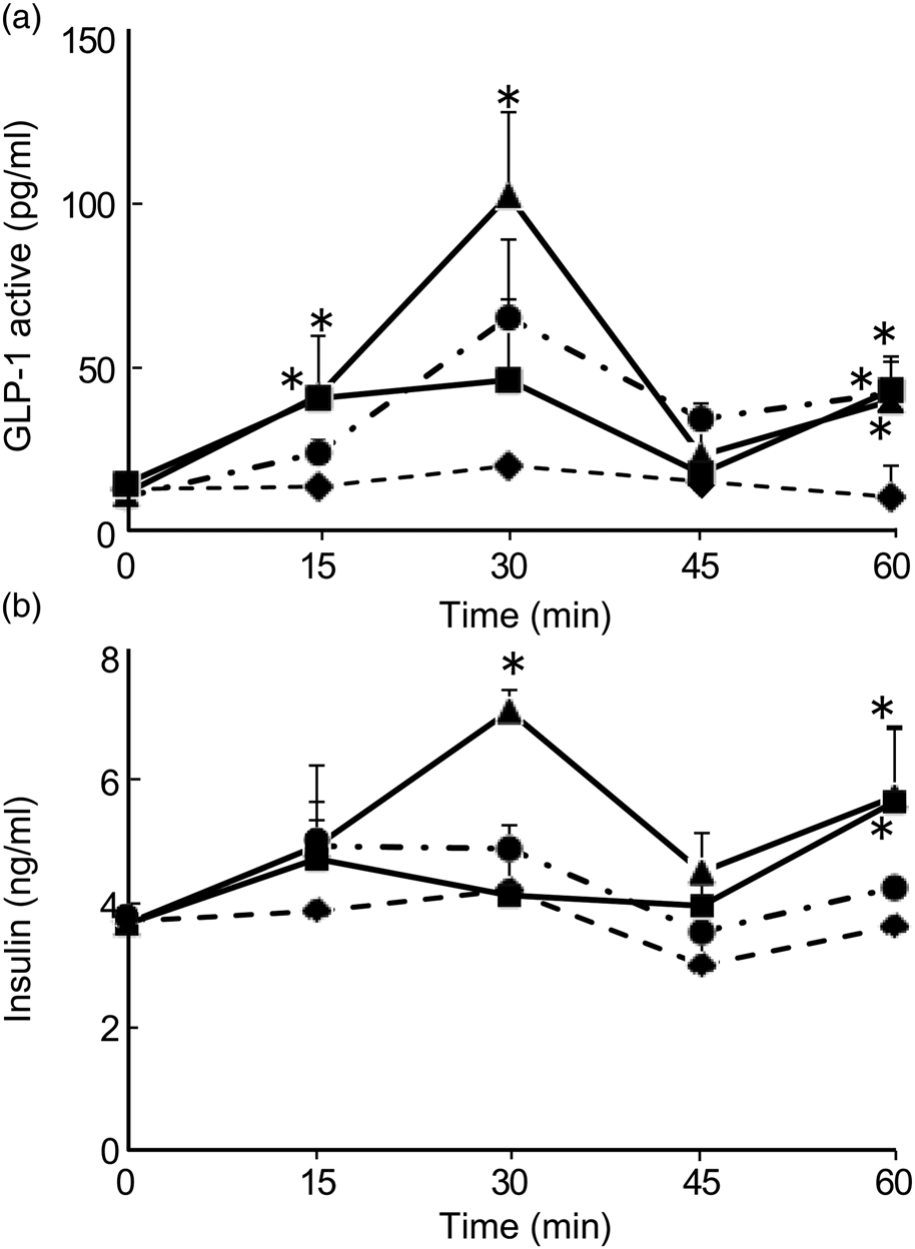
Effect of cacao liquor procyanidin-rich extract (CLPr) and its fractions on the secretion of glucagon-like peptide-1 (GLP-1) (A) and insulin (B) in mouse plasma. Institute of Cancer Research (ICR) mice were dosed orally with 10 mg/kg body weight of the procyanidin fractions (high-degree of polymerisation (DP) fraction (■), low-DP fraction (▲)), CLPr (●) or water (♦) as a vehicle control (5 ml/kg body weight). Plasma GLP-1 and insulin levels were measured at 0 min (before administration of CLPr and its fractions), 15, 30, 45 and 60 min. Values are means (*n* 5), with standard errors represented by vertical bars. * Mean value was significantly different from that of the corresponding control group (*P* < 0·05; Dunnett's test).

**Fig. 7.**
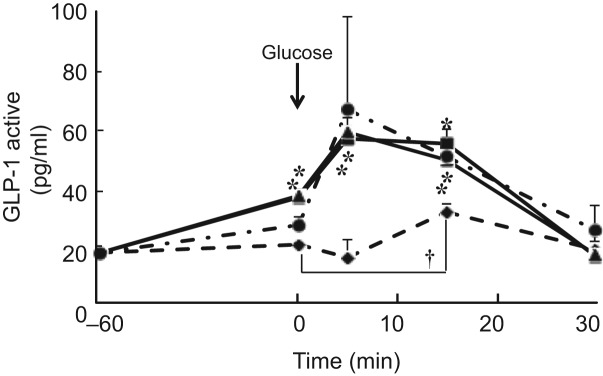
Effect of cacao liquor procyanidin-rich extract (CLPr) and its fractions on the secretion of glucagon-like peptide-1 (GLP-1) in mouse plasma. Institute of Cancer Research (ICR) mice were dosed orally with 10 mg/kg body weight of the procyanidin fractions (high-degree of polymerisation (DP) fraction (■), low-DP fraction (▲)), CLPr (●) or water (♦) as a vehicle control (5 ml/kg body weight). Plasma GLP-1 level was measured −60 min (before administration of CLPr and its fractions), 0 min (immediately before administration of glucose), and 5, 15 and 30 min after glucose administration. Values are means (*n* 5), with standard errors represented by vertical bars. * Mean value was significantly different from that of the corresponding control group (*P* < 0·05; Dunnett's test). † Mean value was significantly different from that of the control group at 0 min (*P* < 0·05; Dunnett's test).

The incretin hormone GLP-1, which is secreted from the gastrointestinal tract, regulates blood glucose levels through the promotion of insulin secretion. To determine the incretin effect of procyanidins on GLP-1 secretion, we measured the plasma GLP-1 level after oral administration of CLPr and its fractions. We found that oral administration of either the high- or low-DP procyanidin fraction increased the plasma GLP-1 level in the absence of glucose load (Fig. 6). Especially, the GLP-1 level in low-DP-given mice at 15, 30 and 60 min, and high-DP-given ones at 15 and 60 min were significantly higher than that in water-given control animals. CLPr increased the GLP-1 level only 60 min after the administration. These results indicated that procyanidin fractions increased GLP-1 secretion in the absence of glucose loading. In the same animals, the insulin levels were also increased: low-DP increased the insulin levels at 30 min and 60 min, while high-DP at 60 min. From these results, low-DP showed the highest efficiency against both GLP-1 and insulin secretions (Fig. 6). Thus, the procyanidin fractions, in particular the low-DP-fraction, induce insulin secretion from pancreatic β-cells through the activation of GLP-1. GLP-1 secretion was also measured after glucose load. Administration of glucose alone showed a slight but significant increase in GLP-1 secretion at 15 min. Both high- and low-DP fractions significantly increased GLP-1 secretion at 5 and 15 min compared with the corresponding water group (which was given glucose solution at 0 min). CLPr also increased GLP-1 secretion at 15 min. These results indicate that CLPr and its fractions have the potential to increase GLP-1 secretion synergistically (at 5 min) or additively (at 15 min) with glucose(Fig. 7).

## Discussion

The pathophysiology of type 2 diabetes mellitus is chronic hyperglycaemia due to impairment of both insulin action and insulin secretion^(^^22^^–^^24^^)^. Current strategies to treat type 2 diabetes mellitus and hyperglycaemia include reducing insulin resistance, and supplementing with exogenous insulin or increasing endogenous insulin production. Recently, many researchers have focused on the incretin hormones, which stimulate insulin secretion from islet β-cells in a glucose-dependent manner after raising plasma glucose concentrations^(^^2^^,^^3^^)^. One of the key incretin hormones is GLP-1, which is secreted from L cells in the gastrointestinal tract and plays a physiological role in glucose metabolism in humans^(^^25^^)^. The potential of polyphenols to activate GLP-1 is reported to be relevant for the treatment of insulin resistance, hyperglycaemia and type 2 diabetes mellitus^(^^7^^–^^9^^)^.

In the present study, we demonstrate that the intake of high- and low-DP procyanidin fractions prepared from cacao liquor extract can prevent postprandial hyperglycaemia through promotion of GLUT4 translocation in skeletal muscle of mice (Figs 1 and 2). The underlying mechanism of these fractions appears to be an increase in GLP-1 activity (Figs 6 and 7) and stimulation of insulin secretion (Fig. 6) in the absence of any nutrient stimulus such as a glucose load. We also confirmed that both fractions activate the insulin-signalling pathway (Figs 4 and 5) and the AMPK pathway (Fig. 3) to promote GLUT4 translocation (Fig. 2). To our knowledge, this is the first report showing a time-dependent change in GLP-1 secretion after a single oral administration of polyphenol in the absence of a glucose load.

Regarding the activation of GLP-1, our findings show that procyanidin-rich fractions from cacao liquor increase GLP-1 secretion in the plasma (Figs 6 and 7). This increase was observed 60 min after administration of all the procyanidin fractions in the absence of glucose load. When glucose was present, an intake of the procyanidin fractions led to an additive or synergistic increase in GLP-1 secretion. Previous results showed that administration of resveratrol for 5 weeks increased glucose-induced GLP-1 secretion in mice^(^^9^^)^. Recently, genistein and daidzein were shown to stimulate GLP-1 in entero-endocrine NCI-H716 cells in high-glucose (20 mm) Krebs–Ringer buffer *in vitro*^(^^26^^)^. These reports suggest that certain polyphenols are able to increase GLP-1 secretion through modulation of the entero-endocrine system. GLP-1 secretion occurs not only in the response to the intake of glucose, but also the intake of fatty acids^(^^27^^,^^28^^)^ and amino acids^(^^28^^)^. The procyanidin fractions used in the present study did not contain glucose, amino acids or fatty acids (data not shown). Thus, certain procyanidins have an incretin effect, although identification of an active compound and its upstream mechanism is still unclear. Further study is needed to clarify these important issues.

With respect to the action of GLP-1, we confirm that high- and low-DP fractions stimulate insulin secretion (Fig. 6) and activate the insulin-signalling pathway (Figs 4 and 5) to promote GLUT4 translocation (Fig. 2) in mouse skeletal muscle. This sequential signal transduction indicates that the intestinal function can control whole-body metabolism. However, there is a discrepancy between the present results and our previous results: our previous report demonstrated that feeding of CLPr for 13 weeks prevented hyperglycaemia through promotion of GLUT4 translocation in the plasma membrane of mouse skeletal muscle by the AMPK-dependent and PI3K-independent pathway^(^^15^^)^. We also showed that the high- and low-DP fractions promoted GLUT4 translocation accompanied by phosphorylation of AMPKα without phosphorylation of Akt in cultured L6 cells^(^^16^^)^. It is likely that the activation of GLP-1 is transient. Indeed our present results indicate that GLP-1 secretion had recovered within 90 min of procyanidin administration even with glucose loading (Fig. 7). In our previous *in vivo* experiments, blood was collected after an 18 h fast^(^^15^^)^. We, therefore, could not observe the activation of the insulin-signalling pathway through the incretin effect. For the *in vitro* experiments, we used L6 myotubes, but not cells derived from the intestine; these cells may lack the ability to secrete GLP-1. Our present findings provide a new possibility that the procyanidin-induced promotion of GLUT4 translocation, which is involved in the prevention of hyperglycaemia, is due to the activation of both the GLP-1-activated insulin-signalling and AMPK pathways. However, the question remains: how do procyanidins activate GLP-1 and AMPK? Although we did not address this question in the present study, we are planning experiments that should give an answer to this important question.

As mentioned above, AMPK is one of the key factors for GLUT4 translocation by procyanidins. Our present study demonstrated that procyanidin-promoted GLUT4 translocation (Fig. 2) is, at least in part, dependent on the AMPK pathway (Fig. 3), in addition to the insulin-signalling pathway (Figs 4 and 5). Previously, polyphenols such as curcumin^(^^29^^)^, epigallocatechin gallate (EGCG)^(^^30^^)^, resveratrol^(^^31^^)^ and anthocyanin^(^^32^^)^ have been reported to promote GLUT4 translocation and glucose uptake in muscle cells and adipocytes through insulin- and/or AMPK-dependent pathways. EGCG is reported to inhibit dexamethasone-induced insulin resistance by activating both the insulin and AMPK-dependent signalling pathways in rat L6 cells^(^^30^^)^. It has also been reported that EGCG improves glucose uptake by activating AMPK and Akt phosphorylation in high-glucose-induced insulin-resistant HepG2 cells^(^^33^^)^. Moreover, we have recently reported that the intake of black tea and pu-erh tea increases GLUT4 translocation by activating both the insulin- and AMPK-dependent signalling pathways in skeletal muscle of mice^(^^34^^)^. These reports indicate that polyphenols are able to promote GLUT4 translocation through both the AMPK- and insulin-signalling pathways, although the upstream events involved in activating these signalling pathways are still unclear.

CLPr does not show a significant GLUT4 translocation (Fig. 2), though it significantly prevented hyperglycaemia estimated by OGTT and IRS-1 phosphorylation in the present study (Figs 1 and 4). In our previous reports, CLPr significantly promoted GLUT4 translocation and its related signalling pathways^(^^14^^)^. This discrepancy may be due to the dose of CLPr: we administered CLPr at 10 mg/kg body weight in the present study, but at 50 and 250 mg/kg body weight in the previous study^(^^14^^)^. The procyanidin concentrations in the high- and low-DP procyanidin fractions were much higher than that of the original CLPr. For example, the low-DP procyanidin fraction contained approximately 3-fold higher levels of monomeric, dimeric and trimeric procyanidins than CLPr, while this fraction contained tetrameric procyanidins (0·23 %) at a level approximately one-quarter of that in CLPr (1·01 %). Both high- and low-DP fractions showed significant effects on GLUT4 translocation, while CLPr showed only a tendency. The most effective procyanidin compound in these fractions remains unclear. We previously demonstrated that cinnamtannin A2 promotes GLP-1 secretion 60 min after administration^(^^10^^)^. In the present study, the high-DP procyanidin fraction slightly more effective than the low-DP one in OGTT (Fig. 1). This difference may explain the difference in the amount of cinnamtannin A2 in this fraction. However, information about the effect of other compounds at the different time points is lacking. Further study is needed to clarify this issue using purified compounds.

Moreover, it should be clarified how and where procyanidins work. Though, we showed that both high-DP and low-DP procyanidin fractions, in addition to CLPr, increased GLP-1 secretion, indicating that L cells in the intestine is one of the targets of procyanidins. However, it remains a question about how procyanidins stimulate secretion of GLP-1. The target molecule in L cells and time for the compound to reach the target molecule are unclear yet. Each procyanidin may have a different residence time in the gastrointestinal tract, and their absorption and metabolic conversion in the tissues including the gastrointestinal tract are not fully understood yet. Recently, we showed absorption and tissue distribution of procyanidin-rich black soyabean seed coat extract in mice^(^^35^^)^ and found that the slight amounts of procyanidin C1 and cinnamtannin A2 appeared in plasma as aglycone, though they mainly exist as conjugated forms in the small intestine. At least, we could not observe an increase in the amount of monomer epicatechin in the same study, indicating that almost the same effects of high-DP and low-DP procyanidin fractions on GLUT4 translocation and its upstream events are not due to the function of monomeric flavan-3-ols such as epicatechin. Furthermore, our previous report using procyanidin compounds^(^^36^^)^ demonstrated that GLUT4 translocation and its upstream events are dependent on the DP of the compound. Therefore, it is still unclear why high-DP and low-DP procyanidin fractions showed the same effects on GLUT4 translocation and its upstream events. Further study is also needed to clarify this issue.

### Conclusion

Procyanidins can prevent postprandial hyperglycaemia through at least two different mechanisms: activation of the insulin-signalling pathway from enhanced GLP-1 secretion and stimulation of AMPK phosphorylation. Both of these mechanisms may coordinately work together to maintain blood glucose homeostasis. Our findings show the potential for procyanidin-rich cacao liquor as an attractive food material for the prevention of hyperglycaemia and type 2 diabetes mellitus.
